# Detecting West Nile Virus in Owls and Raptors by an Antigen-capture Assay

**DOI:** 10.3201/eid1012.040168

**Published:** 2004-12

**Authors:** Ady Y. Gancz, Douglas G. Campbell, Ian K. Barker, Robbin Lindsay, Bruce Hunter

**Affiliations:** * University of Guelph, Guelph, Ontario, Canada;; † Canadian Science Center for Human and Animal Health, Winnipeg, Manitoba, Canada

**Keywords:** West Nile virus, Owl, Raptor, Bird, VecTest, Antigen-capture, PCR, diagnosis, test, Ontario, dispatch

## Abstract

We evaluated a rapid antigen-capture assay (VecTest) for detection of West Nile virus in oropharyngeal and cloacal swabs, collected at necropsy from owls (N = 93) and raptors (N = 27). Sensitivity was 93.5%–95.2% for northern owl species but <42.9% for all other species. Specificity was 100% for owls and 85.7% for raptors.

The emergence of West Nile virus (WNV) in North America has created a demand for reliable, rapid, and economical tests for detecting this flavivirus (family *Flaviviridae*) in a variety of species and sample types. The VecTest (Medical Analysis Systems, Camarillo, CA), a rapid antigen-capture wicking assay, was previously reported to detect WNV in mosquitoes (*1**,**2*) and corvid birds (family *Corvidae)* (*3*).

Little is known about the ability of this test to detect WNV in avian species other than corvids. Low sensitivity of the test when applied to oropharyngeal swabs from dead raptors has been recently reported (*3*). Since the test is largely dependent on the concentration of viral antigen in the analyzed sample (*2**,**3*), the test is more likely to detect WNV in swabs collected from birds that shed large quantities of the virus. Timing of the sample collection with relation to the course of the infection (i.e., acute versus subacute or chronic) is also expected to play a key role, as virus shedding generally is short-lived (*4*).

The objective of this study was to evaluate the usefulness of the VecTest for detecting WNV in oropharyngeal and cloacal swabs from North American owls (family *Strigidae*) and raptors (families *Falconidae*, *Accipitridae*, *Pandionidae*). Based on an observed higher susceptibility of northern versus southern owl species to WNV (*5*), we hypothesized that patterns of virus excretion that influence the sensitivity of this test might differ. Owl species were classified as northern if most of their natural breeding range was north of latitude 48°N, or southern if it was otherwise (*6*).

## The Study

Oropharyngeal and cloacal swabs were collected at necropsy from 87 birds representing 14 species of North American owls, one Eurasian owl, and one falcon that died at the Owl Foundation, Vineland, Ontario (43°10´ N, 79°20´ W) from April 15 to December 25, 2002. This rehabilitation facility had a large-scale WNV outbreak from July to September 2002 (*5*). All birds were kept frozen at –20°C from shortly after the time of death until examination (8–12 months later). Before necropsy, carcasses were allowed to thaw for 24 to 48 h at 4°C.

Oropharyngeal swabs were also collected from 7 owls and 26 diurnal raptors submitted for necropsy to the Canadian Cooperative Wildlife Health Center diagnostic service at the Ontario Veterinary College. These birds were collected from a variety of localities in Ontario and died or were euthanized from August 10, 2002, to July 22, 2003. Most were originally presented to the college's wild bird clinic for veterinary care.

Swabs were collected by rubbing sterile cotton-tipped applicators (provided with the VecTest kit) against the oropharyngeal or cloacal mucosa for 10 s. They were then frozen at –80°C until analyzed (2–6 months for Owl Foundation birds) or tested immediately (wildlife center birds).

For each bird, a full diagnostic necropsy was performed, followed by collection of tissue samples. A pooled sample (about 50 μg in total) of brain, lung, kidney, liver and spleen (foundation birds), or kidney and brain (wildlife center birds), was collected from each bird and tested for WNV RNA by real-time reverse transcription–polymerase chain reaction (RT-PCR) as previously described (*7*). Reported mean C_T_ values (cycles required to reach a fluorescence threshold) are based on 2 to 3 runs per bird using the generic 3´NC primer set (*6*). C_T_ values were available only for Owl Foundation birds.

Before testing, swabs were soaked in 0.5 mL of the grinding solution (provided in the VecTest kit). If initially frozen, swabs were allowed to thaw in the solution for >30 min at room temperature. The test was performed according to the manufacturer's instructions, as previously described (*2**,**3*). All samples were centrifuged for 4 min at 5,200 x *g* before the test strip was inserted. Appearance of even the faintest red line on the test zone at 15 min was considered a positive result (Figure).

**Figure:**
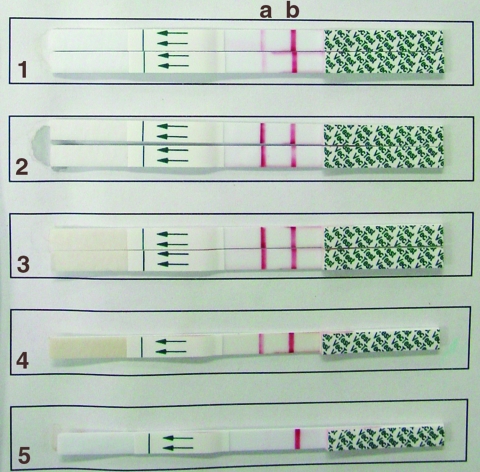
Results of testing by the VecTest assay. Each strip has a test zone (a) and a positive control zone (b). Samples 1–3 were run in duplicate. Note the difference in band intensity between sample 1 vs. samples 2 and 3 (all three are positive). Sample 4 was a positive control and sample 5 was a negative control.

To assess the repeatability of the test, 15 samples were tested simultaneously in duplicates. In two cases, where results were inconclusive because of appearance of uniform red smearing in the test zone, samples were diluted 1:2 in grinding solution and retested. One of those tested positive; the other sample remained inconclusive. For each VecTest kit (50 tests) one positive control (brain and kidney homogenate from an American Crow confirmed to be WNV positive by RT-PCR) and one negative control (water) were tested.

The effect of geographic range and taxonomic group (e.g., owls vs. raptors) on the VecTest sensitivity and on C_T_ values was tested by the Fisher exact test and the Student *t* test, respectively, by using the SAS 8.2 software (SAS Institute Inc., Cary, NC). Based on natural breeding range, the following species were classified as northern: Snowy Owl, Great Gray Owl, Northern Hawk Owl, Boreal Owl, and Northern Saw-whet Owl (for scientific names see Table) These are also the species that had death rates >90% during the 2002 WNV outbreak at the Owl Foundation. All other owl species were considered southern and had death rates of up to 16.7%. These differences and the epidemiology of the outbreak at the Owl Foundation have been described elsewhere (*5*).

**Table:** RT-PCR results and sensitivity of the VecTest assay in 19 species of North American owls and raptors tested for West Nile virus^a^

Species	RT-PCR		VecTest					
			OP swab		Cloacal swab		Combined^b^	
	N	No. positive	N	Sn (%)	N	Sn (%)	N	Sn (%)
Great Gray Owl (*Strix nebulosa*)^c^	19	17	19	100.0	19	100.0	19	100.0
Northern Hawk Owl (*Surnia ulula*)^c^	16	16	16	100.0	15	93.3	15	100.0
Boreal Owl (*Aegolius funereus*)c	13	10	13	80.0	13	90.0	13	90.0
Northern Saw-whet Owl (*Aegolius acadicus*)^c^	12	10	12	100.0	12	100.0	12	100.0
Snowy Owl (*Bubo scandiacus*)^c^	9	9	9	88.9	9	77.8	9	88.9
Great Horned Owl (*Bubo virginianus*)	10	8	10	62.5	4	50.0	4	50.0
Short-eared Owl (*Asio flammeus*)	3	2	3	50.0	3	50.0	3	50.0
Long-eared Owls (*Asio otus*)	3	1	3	100.0	3	100.0	3	100.0
Barred Owl (*Strix varia*)	2	0	2	–	2	–	2	–
Northern Pygmy-Owl (*Glaucidium gnoma*)	1	1	1	0.0	1	0.0	1	0.0
Flammulated Owl (*Otus flammeolus*)	1	1	1	0.0	1	0.0	1	0.0
Tawny Owl (*Strix aluco*)	1	1	1	0.0	1	100	1	100
Eastern Screech-Owl (*Megascops asio*)	2	0	2	–	2	–	2	–
Spotted Owl (*Strix occidentalis*)	1	0	1	–	1	–	1	–
American Kestrel (*Falco sparverius*)	1	0	1	–	1	–	1	–
Red-tailed Hawk (*Buteo jamaicensis*)	19	11	19	36.4	0	–	0	–
Cooper's Hawk (*Accipiter cooperii*)	2	1	2	0.0	0	–	0	–
Sharp-shinned Hawk (*Accipiter striatus*)	4	1	4	100.0	0	–	0	–
Osprey (*Pandion haliaetus*)	1	0	1	–	0	–	0	–
Total	120	89	120	77.5	86	88.4	86	91.4

^a^RT-PCR, real-time reverse transcription polymerase chain reaction; OP, oropharyngeal; N, sample size; Sn, sensitivity. ^b^Results of oropharyngeal and cloacal swab testing were considered in parallel (i.e., the bird was considered positive if one of the two tests was positive). ^c^Northern owl species.

Of 120 birds tested by real-time RT-PCR, 89 (74.2 %) were positive, 30 (25.0 %) were negative, and one was inconclusive (and therefore excluded from further analysis) for WNV. All duplicates gave identical results for each pair.

Of the oropharyngeal swabs tested by VecTest, 71 (59.2%) of 120 were WNV positive (Owl Foundation and wildlife health center birds). When the RT-PCR results were used as the standard, the sensitivity of the VecTest was 77.5% for all birds. The test was significantly more sensitive for owls (85.5%) than for diurnal raptors (30.8%) (p < 0.001), and for northern owl species (95.2%) than for southern owl species (42.9%) (p < 0.001). The difference between raptors and southern owl species was not significant.

The specificity of the VecTest when applied to oropharyngeal swabs was 93.3% for all birds, 100% for owls, and 85.7% for diurnal raptors. Both false-positive results involved Red-tailed Hawks (*Buteo jamaicensis*), one of which had nonsuppurative encephalitis and myocarditis consistent with WNV infection but was WNV negative on PCR and immunohistochemical tests. The positive predictive value (PPV) was 97.2% for all birds, 100% for owls, and 66.7% for diurnal raptors. The negative predictive value (NPV) was 58.3% for all birds, 59.3% for owls, and 57.2% for diurnal raptors.

Of the cloacal swabs tested by VecTest, 61 (71.8%) of 85 were WNV positive (Owl Foundation birds only). Based on the RT-PCR results, the sensitivity of the VecTest when applied to cloacal swabs from all Owl Foundation birds was 88.4%. The test was significantly more sensitive for northern owl species (93.5%) than for other species (42.9%) (p < 0.001). The specificity and PPV of the test were 100%, and the NPV was 66.73% for all Owl Foundation birds.

When the test results of both oropharyngeal and cloacal swabs were considered in parallel (Owl Foundation birds only), 64 (73.6 %) of 87 birds tested positive with an overall sensitivity of 91.4%, specificity and PPV of 100%, and NPV of 72.7%. The sensitivity was 96.8% and 50% for northern and southern owl species, respectively.

C_T_ values were significantly lower (mean 16.78 ± 0.32, n = 62) for northern owl species than for southern owl species (mean 24.56 ± 0.88, n = 8) (p < 0.0001). Birds that were misclassified as negative by the VecTest using either oropharyngeal or cloacal swabs had significantly higher C_T_ values (mean 26.13 ± 4.64, n = 10) compared to all other positive birds (mean 16.25 ± 1.53, n = 60) (p < 0.0001).

## Conclusions

The VecTest proved to be highly sensitive for detecting WNV in oropharyngeal or cloacal swabs from northern owl species, but it showed low sensitivity for samples from southern owl species and raptors. Unlike results with corvids (*3*), cloacal swabs were slightly superior to oropharyngeal swabs. This finding may reflect greater virus shedding from the digestive or urinary systems in northern owl species; however, this hypothesis requires further investigation. Testing both swabs in parallel produced the highest sensitivity. The overall specificity of the VecTest was similar to that reported in corvids (*3*), but it was higher for owls than for raptors (100% vs. 85.7%). Both false-positive samples were from Red-tailed Hawks.

The difference between northern owl species and all other species may reflect higher titers of WNV in the carcasses of northern birds, as indicated also by lower C_T_ values. Northern owl species died significantly earlier during the outbreak period at the Owl Foundation and had high death rates (*5*). These findings suggest differences at the level of the host-virus interactions, possibly affecting virus replication, virus shedding, or the course of the disease (i.e., acute versus chronic). Again, this hypothesis requires further investigation.

The VecTest may be useful as a screening test in birds with suspected WNV infection. However, negative results should be interpreted with caution in light of the test's low sensitivity in some species.
